# Estradiol Metabolism: Crossroads in Pulmonary Arterial Hypertension

**DOI:** 10.3390/ijms21010116

**Published:** 2019-12-23

**Authors:** Stevan P. Tofovic, Edwin K. Jackson

**Affiliations:** 1 Heart, Lung, Blood and Vascular Medicine Institute, University of Pittsburgh, BST E1240, 200 Lothrop Street, Pittsburgh, PA 15261, USA; 2 Department of Pharmacology and Chemical Biology University of Pittsburgh School of Medicine, 100 Technology Drive, PA 15219, USA; edj@pitt.edu

**Keywords:** estradiol metabolism, pulmonary arterial hypertension, 2-methoxyestradiol, CYP1B1, aromatase, sulfatase

## Abstract

Pulmonary arterial hypertension (PAH) is a debilitating and progressive disease that predominantly develops in women. Over the past 15 years, cumulating evidence has pointed toward dysregulated metabolism of sex hormones in animal models and patients with PAH. 17β-estradiol (E2) is metabolized at positions C2, C4, and C16, which leads to the formation of metabolites with different biological/estrogenic activity. Since the first report that 2-methoxyestradiol, a major non-estrogenic metabolite of E2, attenuates the development and progression of experimental pulmonary hypertension (PH), it has become increasingly clear that E2, E2 precursors, and E2 metabolites exhibit both protective and detrimental effects in PH. Furthermore, both experimental and clinical data suggest that E2 has divergent effects in the pulmonary vasculature versus right ventricle (estrogen paradox in PAH). The estrogen paradox is of significant clinical relevance for understanding the development, progression, and prognosis of PAH. This review updates experimental and clinical findings and provides insights into: (1) the potential impacts that pathways of estradiol metabolism (EMet) may have in PAH; (2) the beneficial and adverse effects of estrogens and their precursors/metabolites in experimental PH and human PAH; (3) the co-morbidities and pathological conditions that may alter EMet and influence the development/progression of PAH; (4) the relevance of the intracrinology of sex hormones to vascular remodeling in PAH; and (5) the advantages/disadvantages of different approaches to modulate EMet in PAH. Finally, we propose the three-tier-estrogen effects in PAH concept, which may offer reconciliation of the opposing effects of E2 in PAH and may provide a better understanding of the complex mechanisms by which EMet affects the pulmonary circulation–right ventricular interaction in PAH.

## 1. Introduction

Pulmonary arterial hypertension (PAH), a debilitating progressive cardiopulmonary disease in which increased pulmonary arterial pressure leads to right ventricle (RV) failure and premature death, predominantly develops in women. In the past two decades, data coming from various registries worldwide have confirmed the early observations of female preponderance of PAH made in the 1950s by Doctors Drexler and Wood [1,2]. The female-to-male (F:M) ratio in PAH ranges from 2:1 to 4:1, and even higher (4:1 to 9:1) in PAH associated with connective-tissue diseases (CTD) [3,4,5,6]. In elderly PAH patients, the gender bias disappears (F:M—1.2:1.0) [7], suggesting that female sex hormones are a risk factor for developing PAH. Yet, despite the very early recognition of female preponderance in PAH, for decades, estrogens were held to be protective in PAH. Consequently, the potential pathogenic roles of estrogens in PAH were ignored.

Several factors may have contributed to this lack of interest in the pathogenic roles of estrogens in PAH. First, estrogens are protective in classic rodent models of monocrotaline (MCT)-induced and chronic hypoxia (CH)-induced pulmonary hypertension [8]. We coined the term “estrogen paradox” to describe the discrepancy between female preponderance in humans and the protective effects of estrogens in classic models of pulmonary hypertension [8]. The limitations of classic models of pulmonary hypertension have been reviewed elsewhere [9,10,11]. Studying the role of sex hormones in these models may be of limited value and even misleading [8,12]. Therefore, we refer to the disease in rodents as pulmonary hypertension (PH). In classic models, there is minor involvement of endothelium and absence of occlusive and plexiform lesions (PXL), the hallmarks of vascular remodeling in human PAH. Media hypertrophy, which dominates in these models and is inhibited by estrogens, eventually disappears with the passage of time (MCT-PH) or upon re-exposure to normoxia (CH-PH). This is in contrast to the progressive character of disease in humans. Second, similar to their effects on systemic circulation, estrogens may be protective in the intact pulmonary vasculature, yet in injured endothelium that has been exposed to multiple unknown hits, estrogens may stimulate angioproliferation, promote inflammation, and augment the development of occlusive lesions. Finally, similar to the left heart, female sex hormones may protect the right ventricle (RV) when it is exposed to increased afterload. Recent data in animals, healthy people, and PAH patients support this notion [13,14,15,16]. Recently, the estrogen paradox concept was expanded to encompass the beneficial effects of estrogens concerning RV function and prognosis in PAH [17]. Collectively, we proposed the three-tier effects of estrogens in PAH concept [18], which may help in translating animal data to humans and in elucidating the role of sex hormones in the development, progression, and prognosis of PAH in both women and men.

**Figure 1:**
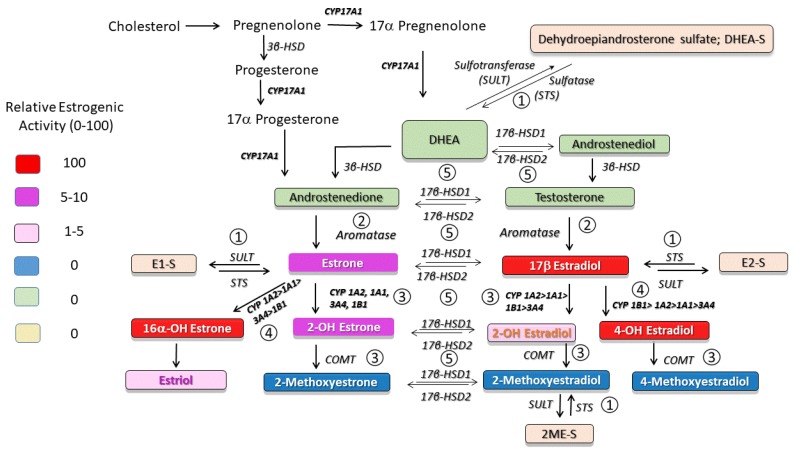
Metabolism of sex steroids. (1) The delicate balance between inactive sulfated sex steroids and sex steroids and their biologically active metabolites/precursors is controlled by the sulfotransferase (SULT)–sulfatase (STS) pathway. (2) Conversion of androgenic precursors to estrogens and intracrine production of estrogens is controlled by the aromatase pathway. (3) The *2-*hydroxylation/methylation pathway of estrogen metabolism produces non-estrogenic metabolites with opposite effects to maternal estrogens. (4) Activation of the 4-Hydroxylation/16α-Hydroxylation pathway leads to the production of highly estrogenic metabolites with proliferative, proinflammatory, and angiogenic properties; (5) The 17β–hydroxysteroid dehydrogenase (17β–HSD) pathway controls the interconversion and delicate intracrine balance between estrogens with high and moderate estrogenic activity, as well as the conversion of biologically inactive 2-methoxyestrone (2ME1) to anti-angiogenic, anti-inflammatory, and pro-apoptotic 2-methoxyestradiol (2ME).

17β-Estradiol (E2) metabolism occurs primarily by oxidation at the C2, C4, and C16 positions of E2 and leads to the formation of metabolites with different biological and estrogenic activity (Figure 1). Since the first report that 2-methoxyestradiol (2ME, a major non-estrogenic metabolite of E2) attenuates the development and progression of MCT-induced PH [12], it has become increasingly clear that E2 and its precursors and metabolites exhibit both protective and detrimental effects in PH [8,19]. Accumulating evidence indicates that, at least in part, both the protective and adverse effects of estradiol in experimental PH are mediated by these metabolites [20,21,22]. In the last decade, evidence has accumulated pointing toward dysregulated metabolism of sex hormones in animal models and patients with PAH. Importantly, these findings suggest the use of estrogen precursors, inhibitors of estrogen production and signaling, and non-estrogenic E2 metabolites for the treatment of PAH.

Previous reviews have focused on the effects of estrogens and their metabolites on pulmonary vascular pathobiology and the development of experimental PH [8,19]. The goal of this review is to provide updated insights into the following: (1) the potential impact that pathways of estradiol metabolism (EMet) may have on PAH; (2) the beneficial and adverse effects of estrogens and their precursors/metabolites in experimental PH and human PAH; (3) the co-morbidities and pathological conditions that, by altering EMet, may influence the development and progression of PAH; (3) the relevance of the intracrinology of sex hormones to vascular remodeling in PAH; and (5) the advantages and disadvantages of different approaches to modulate EMet in PAH. Estrogen signaling in PAH will be only briefly addressed in the context of their metabolism. For a comprehensive review of estrogen signaling in PAH readers are referred to review articles by Lahm and colleagues [23,24].

## 2. Sulfotransferase (SULT)–Sulfatase (STS) Pathway

The synthesis and breakdown of estrogens are complex (Figure 1). Dehydroepiandrosterone (DHEA) serves as pivotal precursor for E2 biosynthesis. DHEA is converted to DHEA sulfate (DHEA-S) by sulfotransferase (SULT), and DHEA-S can be converted back to DHEA by steroid sulfatase (STS). The balanced effects of STS and SULT [25] control the ratio of DHEA to DHEA-S. Until recently, sulfated steroids including DHEA-S were considered inert circulating metabolic products that were incapable of binding to and activating nuclear receptors. Circulating sulfated steroids are hydrophilic and they are readily available for renal excretion; to produce local/cellular effects they would require organic anion transporters (OATPs) for active transport into cells. Notably, OATPs are expressed in multiple cells and tissues, including endothelial and inflammatory cells and lungs [26,27]. Besides DHEA-S, other important hormone substrates for STS include estrone sulfate (E1-S) and estradiol sulfate (E2-S). Therefore, the sulfatase pathway represents a major intracrine route for regenerating biologically active estrogens (Figure 1). On the other hand, in target cells, the cytosolic enzyme SULT, by conjugating a sulfonate group to estrogens, inhibits E2 binding to and activity at estrogen receptors (ER) and augments the renal excretion of estrogens [28]. Thereby, SULT and STS are negative and positive regulators of the estrogen activity, respectively [29]. Sulfotransferase also catalyzes sulfation of 2ME, and increased SULT activity correlates with diminished anti-mitogenic effects of 2ME in various cancer cell lines [30]. Hence, SULT may modulate E2 signaling, regulate E2 intracrinology and inactivate biologically active E2 metabolites [30].

DHEA is synthesized in both postmenopausal women and men by the adrenal cortex and by peripheral conversion from circulating DHEA-S, and by ovaries and placenta in premenopausal women [31,32]. Adrenal production of DHEA takes place only in humans and primates, whereas rodents and other species have very low levels of DHEA that are produced by other organs. DHEA and its downstream metabolite androstenedione are the most abundant circulating steroids. The conversion of DHEA to androstenedione is facilitated by 3β-HSD, and in peripheral tissues, circulating androstenedione can be converted by aromatase to estrone [33,34].

### 2.1. SULT–STS Pathway in PAH

Although the roles of sulfatase, SULT, 3β-HSD, and androstenedione in PAH are unknown, a growing body of evidence supports important roles for DHEA in PAH (Table 1). At the cellular level, DHEA reduces hypoxia-induced accumulation of hypoxia-inducible factor 1α (HIF)-1α in human pulmonary artery endothelial cells (hPAECs) [35] and stimulates NO release in bovine endothelial cells (ECs) [36]. In animals, preventive and/or rescue treatments with DHEA or DHEA-S are effective in CH-PH [37,38,39], MCT-PH [40,41], fetal pulmonary circulation in sheep [42], the rat model of PH in infants [43], and in Sugen 5416 + hypoxia (Su-Hx)-induced PH [44]. DHEA also attenuates PH in patients with chronic obstructive pulmonary disease [45].

Recently, Ventetuolo et al. [46] reported that lower DHEA-S, greater E2 levels, and increased E2- to-testosterone ratios correlated with shorter 6-min walking distances (6MWD) and were associated with a greater risk of PAH in men. Furthermore, DHEA-S levels were inversely associated with right atrial pressures and pulmonary vascular resistances. Similar changes in sex hormones were associated with worse hemodynamics, functional class, shorter 6MWD, and greater risk of death in post-menopausal women with idiopathic-, CTD-, or heart disease-associated PAH [47]. Altogether, the reported pattern of hormonal changes in men and postmenopausal women with PAH points toward increased activity of sulfatase and increased downstream aromatization of androgens.

### 2.2. Future Directions and Clinical Implications

The above data suggest potential therapeutic effects of DHEA in PAH. However, although increased estrogen levels by pharmacological doses of DHEA may be beneficial with respect to the RV, they may exacerbate pulmonary angioproliferative remodeling and may increase the risk of sex hormone-dependent cancer in women with PAH. Although DHEA is an over-the-counter supplement with no major acute side effects, the long-term safety of DHEA in patients with PAH is unknown. Therefore, the long-term safety of high doses of DHEA (and the effects of subsequently elevated circulating estrogens) should be further investigated, at least in the Su+Hx PH model.

## 3. Aromatase Pathway

The aromatase pathway is a major biochemical system for the production of E2. Aromatase is encoded by the CYP19A1 gene and converts testosterone to E2 and androstenedione to E1. This conversion takes place in the ovaries and in peripheral tissues, and the aromatase pathway is a major source of extra gonadal estrogen production in postmenopausal women and men [66]. Compared to other CYP450 encoding genes, the CYP19A1 gene is unique, as it contains a number of untranslated exons that occur in aromatase transcripts in a tissue-specific fashion, due to differential splicing that gives rise to tissue-specific promoters [67,68]. Therefore, the regulation of aromatase via alternatively used promoters can differ in various tissues/organs in health and disease, and might be activated/inhibited by a host of local factors (including estrogens and their metabolites) [66,67]. Relevant to PAH, human endothelium expresses an entire aromatase–estrogen–E2 receptor system.

### 3.1. Aromatase in PAH

Early studies reported 2–3-fold greater aromatase activity in male than in female neuroendocrine tissues [67,69], whereas the most recent ones suggest increased aromatase expression in female human pulmonary artery smooth muscle cells (hPASMCs) and healthy and Sugen 5416 + Hypoxia treated female murine lung [55], but decreased lung aromatase expression in female MCT-PH rats [48]. These reported discrepancies may reflect tissue/disease specific regulation of aromatase. Similar to the increased aromatization of androgens in postmenopausal women and men with PAH [46,47], in patients with portopulmonary PAH, irrespective of sex, elevated aromatase activity and plasma E2 levels are associated with increased risk of PAH [58]. Significantly, E2 augments aromatase activity, and by increasing aromatization of androgens may augment its own production [70]. In contrast, 2-ME inhibits both basal- and TNFα-stimulated aromatase activity [71], and thereby may reduce extra-gonadal synthesis of estrogens.

In humans, the third-generation aromatase inhibitor (ARO-I) anastrozole reduces not only E2, but also E1 and E1S levels, suggesting indirect inhibition of local E2 production via 17β–HSD and sulfatase pathways. In hypoxic mice and Su-Hx rats, anastrozole reduces E2 levels and attenuates PH in female, but not in male animals [55]. Moreover, in combination with fulvestrant (selective estrogen receptor degrader) anastrozole reverses PH in BMPR2 mutant mice [57]. Finally, metformin, a first line anti-diabetic drug, inhibits aromatase expression [72] and has beneficial effects in angioproliferative PH in female rats [56]. Collectively, the experimental and observational data point toward a potential use of ARO-I in PH. Indeed, in a recent pilot phase 2 clinical trial in postmenopausal women and men with PAH, Kawut et al. reported that 12-week treatment with anastrozole reduced serum E2 levels by 40% and E1 levels by 70%, and significantly increased 6MWD [59].

### 3.2. Aromatase Inhibition and RV Function in PAH

Although experimental and initial clinical data with anastrazole are encouraging, the benefits and potential harm of long-term inhibition of E2 synthesis are unknown. ARO-I is well tolerated by cancer patients, yet reduced E2 production by ARO-I could deprive the RV of the protective effects of estrogens, in particular in PAH, where the RV is exposed to increased workload. In this regard, both exogenous and endogenous E2 protect RV function and structure in experimental PH [15,62]. Data from the Multi-Ethnic Study of Atherosclerosis (MESA study) clearly indicate that high E2 levels benefit RV function [14], and in ovariectomized (OVX) Su-Hx rats, exogenous E2 protects RV function by preserving mitochondrial content and oxidative capacity [60]. Preliminary data in female Su-Hx rats (published in abstract form [54]) suggest that despite reducing PH and the number of occlusive lesions, anastrozole does not reduce RV hypertrophy, as would be expected from reduced occlusive remodeling and reduced afterload. Finally, although anastrazole on average had no effects on echocardiographic parameters and biomarkers of RV function in the proof-of-concept study on PAH, anastrazole had varying effects on individual patients’ RV function, with some experiencing worsening in RV function [59].

### 3.3. Future Directions and Clinical Implications

The ongoing large phase 2 study (NCT03229499) should provide data on the short-term efficacy/safety of anastrazole in PAH. Yet, the long-term effects of anastrozole on the progression of disease, RV function, and mortality will remain unknown. Studies in the Su+Hx model focused on the interaction between anastrozole and progression of RV dysfunction could help address the potential long-term negative impact of anastrazole on RV function. Based on our preliminary data (published abstract [73]), we suggest studies of anastrazole in female rats exposed to escalating doses of SU5416 (20-50-100 mg/kg) that rapidly (within five to six weeks) produce an increasing level of RV dysfunction/failure.

## 4. 2-Hydroxylation/2–Methylation Pathway

**Figure 2:**
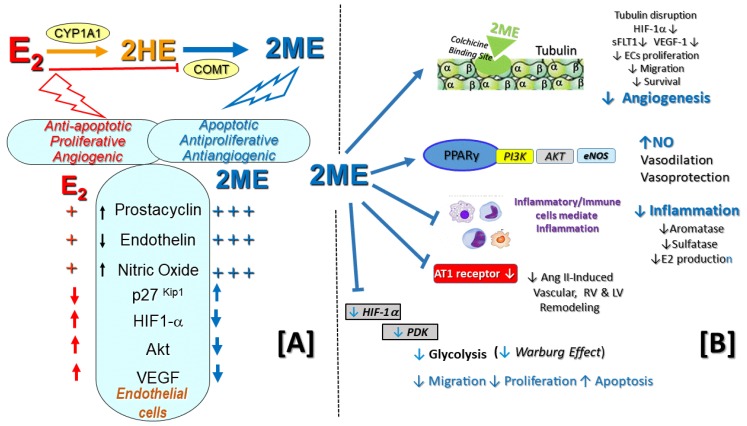
Putative mechanisms of action of 2ME relevant to PAH. 2ME could be viewed as a new disease modifier in PAH. (**A**) In injured endothelium in PAH, 2ME behaves as a biological antagonist of estradiol (E2). 2ME and E2 have opposite effects on key regulators of angioproliferation (p27Kip1, AKT, HIF1-α, VEGF) and 2ME is a more potent modulator of prostacyclin, endothelin, and nitric oxide synthesis/release than E2. (**B**) 2ME binds to the colchicine-binding site, disrupts the microtubule cytoskeleton, downregulates HIF1-α and inhibits angiogenesis and glycolysis. +++ vs. +, stronger effect of 2ME vs. E2; ↑ and ↓: E2 (red) and 2ME (blue) related increase/stimulation and decrease/inhibition, respectively. [B] black ↑ and ↓: subsequent and/or indirect effects of 2ME; blue arrows: binding of or stimulation by 2ME; blue T arrows: inhibition/down-regulation by 2ME.

Once formed, E2 can be converted to biologically active metabolites via the sequential steps of hydroxylation mediated by multiple CYP450 enzymes (Figure 1 and Figure 2), followed by methylation of hydroxyl groups catalyzed by catechol-*O*-methyl transferase (COMT). The 2-hydroxylation/2-methylation pathway is a major metabolic process that, by some estimates, accounts for ~50% of E2 metabolism. This process largely takes place in the liver, where E2 is mainly metabolized by CYP1A1/1A2/1B1 and CYP3A4 to 2-hydroxyestradiol (2HE) and, to a lesser degree (~5%), by Cyp1B1 to 4-hydroxyestradiol (4HE). Catechol estrogens have various levels of estrogenic activity and are converted by COMT to methoxyestradiols. In addition to hepatocytes and cancer cells, the conversion of E2 to downstream 2HE and 2ME also takes place in cardiovascular compartments [65]. The 2-hydroxylaton/methylation pathway produces non-estrogenic, anti-proliferative, anti-angiogenic, and anti-inflammatory metabolites (2HE and 2ME) [74]. Notably, the inhibition of CYP1A1 or COMT attenuates or blocks the antimitotic effects of E2 [74]. Furthermore, the inhibition of vascular remodeling in MCT-OVX rats by E2 and inhibition of carotid artery remodeling by 2ME are associated with reduced p-Akt and increased COX-2 expression [48,75].

### 4.1. 2-Hydroxylation/2-Methylation Pathway in PAH

Currently, the impact of this metabolic pathway in PAH are unknown. Yet, the above findings suggest that in vivo, the anti-proliferative effects of E2 in the vascular compartment may be at least in part mediated by the metabolism of E2 to 2ME (Figure 2). Indeed, as summarized in Table 2, 2ME not only reverses MCT-PH in male rats, but also, at least in part, mediates the beneficial effects of E2 in OVX rats with MCT- and bleomycin-induced PH and lung fibrosis [12,20,21]. 2ME is also protective in hypoxia-induced PH [76,77,78] and 2ME has synergistic therapeutic effects with sildenafil and bosentan to ameliorate MCT-induced PH, inflammation, and vascular remodeling [79]. Compared to E2, in ECs 2ME is a more potent modulator of NO, prostacyclin, and endothelin synthesis [8,74,80], three major pharmacological targets in PAH. The 2ME-induced NO release in ECs is mediated via activation of the PPARγ/PI3K/Akt pathway and results in vasodilation [81]. 2ME could be viewed as a mediator of E2′s beneficial effects in PAH. The high proliferative state of human leiomyoma cells (hLCs) is characterized by doubled ERα signaling, which is inherently regulated by microtubule dynamics [82]. Notably, COMT over expression or treatment with 2ME stabilizes microtubules, attenuates E2-induced proliferation, inhibits ERα signaling, and reduces HIF-1α and aromatase expression in hLCs [83,84]. That COMT and 2ME may induce similar changes in highly proliferative hPASMCs overexpressing aromatase and pulmonary artery endothelial cells (PAECs) overexpressing HIF-1α [55] is an attractive possibility that needs to be examined.

### 4.2. Other Effects of the 2-Methylation Pathway Relevant to PAH

There are several aspects of the 2-methylation metabolic pathway that are relevant to PAH. In this regard, cumulating evidence suggests that the renin–angiotensin system (RAS) contributes to the development of PAH [85,86,87]. Remarkably, COMT deficiency is associated with increased sensitivity to angiotensin II (Ang II), and in COMT -/- and CYP1B1 -/- mice that have reduced 2ME production, 2ME treatment abolishes hypersensitivity to and vascular injury induced by Ang II [88,89]. Of note is that 2ME via activation of the G-protein-coupled estrogen receptor (GPER) downregulates Ang II type 1 receptor [81,82,90,91]. Of relevance to PAH, 2ME attenuates Ang-II induced vascular and cardiac remodeling and fibrosis and isoproterenol-induced (Ang II-mediated) RV and LV hypertrophy and fibrosis [92].

Another aspect of the 2-methylation metabolic pathway relevant to PAH is its effects on obesity and the metabolic syndrome (MS), two established risk factors for PAH [93,94,95,96,97,98]. Experimental and clinical data link reduced COMT activity and 2ME levels to the development of obesity and insulin resistance [99,100]. Moreover, a low-activity variant of COMT (val/met 158) may contribute to MS [99], whereas the high-activity form of COMT (rs4680) is associated with lower HbA1c and protection from type 2 diabetes [101]. Also, both genetic and pharmacologically-induced deficiency of COMT exacerbates high-fat diet (HFD)-induced insulin resistance in mice [100]. In COMT-deficient mice on HFD, 2ME, which shares structural similarity with PPARγ ligands and acts as a PPARγ agonist [75,89], increases glucose tolerance, insulin secretion, and adenosine monophosphate kinase (AMPK) phosphorylation in the liver and islet cells [100].

Yet another aspect of the 2-methylation pathway potentially relevant to PAH is the fact that E2 regulates COMT activity and possibly its downstream conversion to non-estrogenic 2ME. On this subject, COMT is highly expressed in human and rat lungs [102,103]; men have higher hepatic COMT activity than women [104]; in vitro E2 decreases COMT transcription, activity, and protein levels [105,106]; exposure to E2 reduces hepatic COMT activity in rats [107]; and by antagonizing E2, tamoxifen increases COMT activity in peripheral tissues [108]. 

Additional biological effects of 2ME that may be of relevance to PAH are summarized in Figure 2. It should be reemphasized that in cardiovascular and renal cells, 2ME does not interact with classical ERα and ERβ nuclear receptors [74]. Cardiac and vascular protection of E2 has been linked to the activation of GPER located on the cell membrane [109]. More importantly, the activation of GPER reverses PH-related cardiopulmonary dysfunction and exercise intolerance in both male and female MCT-PH rats [110,111]. In this regard, 2ME binds the membrane GPER and, via GPER-mediated transactivation of EGFR and ERK1/2 phosphorylation, downregulates AT1 receptor expression [90,91]. Yet, the antimitogenic effects of 2ME in microglia cells and vascular smooth muscle cells (VSMCs) are independent of GPER or nuclear E2 receptors [112]. Whether cardiovascular protective effects of 2ME are mediated by GPER remains to be determined. Inhibition of the HIFα–VEGF axis is one of the most prominent effects of 2ME. This action is highly relevant to PAH and is discussed further in Section 7.

### 4.3. Future Directions and Clinical Implications

The role of COMT and the effects of 2ME in patients with PAH are unknown. Nonetheless, a growing body of evidence strongly supports further investigation of the E2–COMT–2ME interrelationships in experimental angioproliferative PH and PAH patients. This evidence includes: (1) the modulation of RAS activity by COMT and 2ME; (2) the role of COMT and 2ME in insulin resistance and the metabolic syndrome; (3) the effects E2 on COMT activity; and (4) the opposite effects of 2ME and E2 on angiogenesis (see Section 7).

## 5. 4- and 16α-Hydroxylation Pathways

The oxidation of estradiol at C4 by CYP1B1 leads to the production of 4-hydroxyestradiol (4HE), a catechol estradiol with reactive oxygen species (ROS)-dependent, but ER-independent, carcinogenic effects. 4HE can undergo methylation by COMT to 4-methoxyestradiol (4ME), oxidation to quinones, or dehydrogenation (by 17β–HSD) to 4-hydroxyestrone (4HE1; Figure 1). The oxidation of E2 and E1 at C16 (by CYP1A1, CYP1A2, CYP2C8, and CYP3A) produces 16α-hydroxylestradiol (16αHE; estriol) and 16α-hydroxyestrone (16αHE1), respectively. Estriol is a weak estrogen produced in abundance during pregnancy and is further oxidized by 17β–HSD2 to 16αHE1, a highly estrogenic metabolite that covalently binds to ERs and induces prolonged ER activation [121]. 

### 5.1. Role of CYP1B1 in PAH

New data obtained over the last decade suggest a major pathogenic role for 16αHE1 and Cyp1B1 in PAH. Both human and animal data coming largely from Vanderbilt [120,122,123,124], Glasgow [119,125,126], and Penn State Universities [14,127] clearly point toward a pathogenic role for CYP1B1 with regard to the risk of PAH in humans and toward a pathogenic 16αHE1–BMPR2 interaction in experimental PH. What is less clear is: (1) whether the 16α-hydroxylation pathway (i.e., 16αHE1) is a major pathogenic factor in human PAH; (2) whether CYP1B1 activity is directly linked to significant production of 16αHE1; and (3) whether the reported effects are related directly to CYP1B1 modulation of EMet versus the effects of CYP1B1 unrelated to EMet. What has been overlooked is: (1) differences in CYP1B1 activity in humans versus rodents; (2) the promiscuity of CYP1B1 for various non-estrogenic substrates (and thereby the effects of CYP1B1 inhibitors unrelated to estrogen metabolism); (3) the lack of direct/causative evidence about CYP1B1 effects on 16αHE1 production; and (4) limitations of interpreting urinary estrogen data (2HE1/16α-HE1 ratio) to draw associations between CYP1B1 activity, 16αHE1 production, and their pathogenic roles in PAH.

### 5.2. Divergent Effects on E2 and 2ME on CYP1B1 Activity

In humans, CYP1B1 is constitutively expressed in the lung and other tissues, including VSMCs and ECs [128], but not hepatocytes. Initial data indicated that human CYP1A1 predominantly controls 2HE and CYP1B1 4HE formation [129]. Importantly, in contrast to human CYP1B1, which favors a four-fold greater production of 4HE versus 2HE, rat CYP1B1 favors a two-fold production of 2HE compared to 4HE [130]. Notably, E2 and 2ME have divergent effects on CPY1B1 activity. Estradiol is not only a substrate for CYP1B1, but also (via ERα) is a transcriptional activator of CYP1B1 [131]. In contrast, in vitro 2ME exerts feedback inhibition on CYP1B1 activity [132] and inhibits aryl hydrocarbon receptor-mediated induction of CYP1B1 and downstream production of reactive metabolites. In vivo 2ME significantly inhibits CYP1B1 expression, reduces mid-chain hydroxyeicosatetraenoic acid (HETE) production, and attenuates pressure overload-induced cardiac remodeling [133].

### 5.3. Role of CYP1B1 and Estrogens in Arachidonic Acid Metabolism

**Figure 3:**
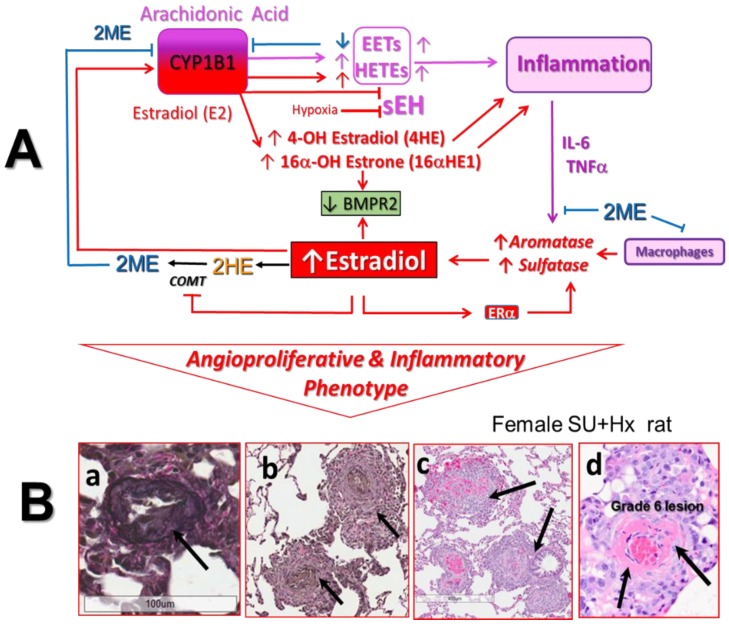
(**A**) Dual metabolic activity of CYP1B1 and an inflammation-instigated estradiol (E2)-feed forward mechanism that involves sulfatase, aromatase, E2, and BMPR2 and results in the development of an angioproliferative and inflammatory phenotype and increased risk of PAH in women. E2 and 2-methoxyestradiol (2ME) have opposite effects on CYP1B1 activity and E2 and arachidonic acid (AA) metabolism, which leads to the production of proinflammatory E2 and AA metabolites. Red arrows: estrogens related increase/stimulation or red T arrows reduction/inhibition of enzyme expression/activity, E2 and AA metabolite production and inflammation, and BMPR2 down-regulation. Magenta arrows: CYP1B1 activation and related AA metabolism result in the production of proinflammatory AA metabolites and up-regulation of E2 producing enzymes; Blue T arrows: 2ME inhibits CYP1B1 activity and the production of AA metabolites and proinflammatory cytokines induced by estrogen-producing enzymes. ↑ and ↓: estrogens (red) CYP1B1 (magenta) and 2ME (blue) related increase and decrease, respectively; BMPR2 = bone morphogenetic protein receptor type 2; COMT = catechol-*O*-methyltransferase; CYP = cytochrome p450 enzymes; EETs = epoxyeicosatrienoic acids; HETEs = hydroxyeicosatetraenoic acids; she = soluble epoxide hydrolase. (**B**) Compared to males, female SU + Hx PH rats exhibit (indicated by arrows) exacerbated angioproliferation, i.e. occlusive lesions (**a**) and perivascular inflammation (**b**,**c**), and sporadically develop necrotizing arteritis i.e., grade 6 lesions (**d**).

In addition to the oxidation of xenobiotic and production of catechol estrogens, CYP1B1 is also involved in the metabolism of arachidonic acid (AA), melatonin, and retinoid. Of particular relevance to PAH is CYP1B1 lipoxygenase-like activity, which facilitates AA metabolism into mid-chain hydroxyeicosatetraenoic acids (HETEs) and epoxyeicosatrienoic acids (EETs; Figure 3) [134]. For example, CYP1B1 metabolizes AA into 12- and 20-HETEs, which stimulate VSMC growth [135]. HETEs also promote hypoxic pulmonary vasoconstriction and vascular remodeling and inhibit apoptosis of PAVSMCs [136,137,138]. In vivo inhibition of CYP1B1 reduces HETE formation and doxorubicin-induced cardiac fibrosis/failure [139]. In PAVSMCs and pulmonary artery ECs (PAECs), both EETs and HETEs have inflammatory, mitogenic/angiogenic, and vasoconstrictive effects and are implicated in the development of hypoxic PH [136,140]. Furthermore, in PAH patients, there is increased production of HETEs that correlates with poor prognosis [141]. 

What has been overlooked is the opposite effects that E2 and 2ME may have on HETE and EET production/metabolism. In this regard, E2 via ERα stimulates CYP1B1 activity and may increase HETE and EET production [142,143]. In contrast, 2ME inhibits CYP1B1 activity and reduces HETE formation and cardiac remodeling in rats with pressure overload-induced cardiac hypertrophy [114]. Moreover, E2 inhibits the expression/activity of soluble epoxide hydrolase (sEH), a key enzyme in EET metabolism [144]. Several studies linked low sEH activity to the pathophysiology of PAH: (1) E2-, genetic-, and pharmacologically-induced downregulation of sEH (and increased pulmonary EET) potentiates hypoxic pulmonary vasoconstriction [145,146,147]; (2) lungs from PH patients express no/little sEH; (3) hypoxia decreases the expression of sEH; and (4) when exposed to hypoxia, sEH KO mice exhibit exacerbated pulmonary vascular remodeling [147]. It seems that E2 plays a role in female-specific (physiological) downregulation of sEH, as suggested by the much higher sEH activity in male and OVX mice compared to intact female mice [148,149]. The E2 regulated sexually dimorphic expression of sEH and bioavailability of pathogenic EET and HETE in the pulmonary circulation could explain the female preponderance of PAH [142].

### 5.4. Future Directions and Clinical Implications

The fact that E2 in a sex-specific manner may regulate the bioavailability of pathogenic EET and HETE in the pulmonary circulation suggests an intriguing explanation for the female preponderance of PAH. However, this hypothesis requires further investigation. Thus far, the roles of CYP1B1 and estrogens in AA metabolism in PAH have not been studied. Any further investigation and discussion regarding the pathogenic roles of CYP1B1 in PAH should address the effects of E2 on CYP1B1 and sEH activity and related EET and HETE production and metabolism. The latter is also relevant to the assessment of the claimed “major role” of CYP1B1 in production of 16αHE1, a “major” pathogenic E2 metabolite in PAH. The dual-metabolic activity of CYP1B1, proinflammatory AA metabolites, and inflammation-instigated feedforward mechanism of E2 production (Figure 3) may shift E2 metabolism toward the production of pathogenic 16αHE1 and significantly reduce the 2HE1/16α-HE1 ratio (processes documented in patients with autoimmune inflammatory diseases). Accordingly, the 2HE1/16α-HE1 ratio should not be used to draw associations between CYP1B1 activity, 16αHE1 production, and their pathogenic role in PAH.

## 6. 17β-Hydroxysteroid Dehydrogenase Pathway

**Figure 4:**
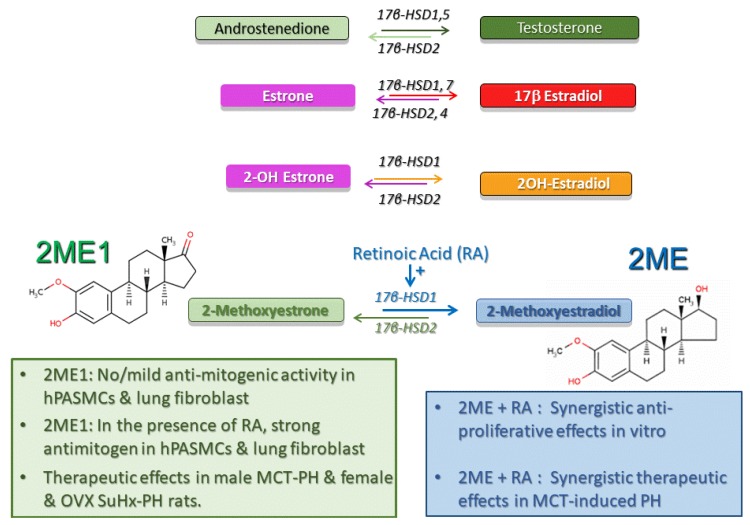
The 17β-HSD pathway controls the interconversion and delicate intracrine balance between estrogens with high and moderate estrogenic activity, as well as the conversion of biologically inactive 2-methoxyestrone (2ME1) to anti-angiogenic, anti-inflammatory, and pro-apoptotic 2-methoxyestradiol (2ME). RA = retinoic acid, an inducer of 17β-HSD1; hPASMCs = human pulmonary artery smooth muscle cells; MCT = monocrotaline; OVX = ovariectomy.

17β-Hydroxysteroid dehydrogenases (17β-HSDs) catalyze stereospecific oxido-reduction reactions at position 17 of estrogens and androgens and play a key role in the last step of activation and first step of degradation of estrogens and androgens (Figure 4). Type-1 17β–HSD (17βHSD-1) catalyzes the reductive transformation of less estrogenic estrone (E1) to E2 and this process takes place not only in cancer cells, but also in vascular smooth muscle cells [150,151]. The oxidative type-2 17β-HSD (17βHSD-2) converts E2 to E1. Additionally, 17β-HSD types 7 and 12 catalyze the production of E2, types 3 and 5 contribute to testosterone production, and types 4, 8, and 10 oxidize E2 and testosterone to less active forms of these steroids [152].

### 6.1. 17β-HSD Pathway and 2ME Disposition

The impact of the intracrinology and cellular disposition of estrogens and their metabolite in the pulmonary vasculature and PAH is unknown. However, it seems that the 17βHSD pathway plays a critical role in 2ME inactivation. 2ME is extensively metabolized by 17βHSD-2 to 2-methoxyestrone (2ME1), a metabolite largely considered to be biologically inactive. 2ME exhibits highly antimitogenic effects in both ER+/ER- human breast cancer cell lines with low 17βHSD-2 activity, whereas cells with high 17βHSD-2 activity are selectively resistant to the antimitotic effects of 2ME [138,153]. Related to 2ME disposition, the very fast uptake contributing to high cellular concentrations (>15 μmol/L) of 2ME takes place in highly proliferative and E2 sensitive MCF7 cells [154], and similar fast uptake and high cellular concentrations should be expected for more lipophilic 2ME1. Furthermore, in rats with MCT-induced PH, increases in the dose of 2ME do not additionally reduce PH and RV hypertrophy, yet additionally inhibit media remodeling and inflammation [113]. Finally, continuous use of large oral doses of 2ME in patients with various types of cancer results in minimal 2ME urinary excretion, but yields high plasma levels of 2ME1 (10–20-fold higher than 2ME) [155,156,157]. The above data indicate that 2ME–2ME1 interconversion takes place in humans and suggest that the cellular disposition of 2ME and 2ME1 may play a significant role in the biological and pharmacological effects of 2ME.

### 6.2. 17β–HSD Pathway in Experimental PH

The above discussion and data in female cancer patients point toward a key role of the 17β–HSD pathway in intracrinology. Thus, 17β–HSD likely modulates the biological effects of estrogens and their metabolites by altering their cellular disposition. Our preliminary data published in abstract form [115,116,117] (manuscript in preparation) support the notion that this may be also the case in PH. For example, in vitro, in contrast to 2ME, 2ME1 has only mild antimitogenic effects at high pharmacological concentrations (10 μM) [116]. However, in the presence of retinoic acid (RA, a 17βHSD-1 inducer), 2ME1 strongly inhibits the growth of human pulmonary artery smooth muscle cells (PASMCs) and lung fibroblasts, suggesting that the 2ME–2ME1 interconversion (2ME ↔ 2ME1) takes place in cells involved in vascular remodeling in PH [116]. In vivo, in MCT-induced PH in male rats, 2ME and RA have synergistic therapeutic effects; yet in vitro (in the absence of 2ME1), RA does not influence the marked antimitogenic effects of 2ME [115]. In vivo, in male MCT rats, rescue treatment with 2ME1 reduces PH, media remodeling, and inflammation (influx of ED1 cells) [116]. Finally, in intact and OVX female Su-Hx rats that exhibit severe angioproliferative PH and sporadically develop necrotizing arteritis, rescue treatment with 2ME1 reduces PH, RV dysfunction and remodeling, and the number of occlusive lesions and prevents the development of grade 6 lesions [117].

### 6.3. Future Directions and Clinical Implications

Currently, whether the 17β–HSD metabolic pathway plays a role in PAH is unknown. Yet, available data suggest that the 17β–HSD pathway controls the intracellular disposition of estrogens and their metabolites, and thereby determines the relative concentrations of steroids with low versus high estrogenic activity and “good” versus “bad” pharmacological effects. This warrants further investigation into the participation of 17β–HSD in PAH. Studying the effects of selective 17β–HSD1 inducers and/or 17β–HSD2 inhibitors on EMet (i.e., intracrinology of E2 and 2ME) and on development/progression of disease in angioproliferative PH should be part of this line of research.

## 7. Angiogenesis, Metabolic Reprograming, and Estradiol Metabolism in PAH

Vascular lesions in patients with severe PAH are characterized by the existence of two types of EC phenotypes: (1) normal quiescent apoptosis-sensitive ECs located in peripheral areas, which have a high expression of p27kip1 (a marker of low growth); and (2) highly proliferative apoptosis-resistant cells in the central core of the vascular lesion, which have low expression of p27kip1 and increased expression of HIF-1α, VEGF protein, and VEGF-2 receptor [158,159]. The hypoxia-activated HIF-1α–VEGF axis plays a key role in vascular reactivity/remodeling and angiogenesis [160]. In severe PAH in humans, HIF-1α is overexpressed in obliterative endothelial lesions [158] and in experimental PH, a time-dependent increase in HIF-1α levels/expression correlates with the development of disease and vascular and RV remodeling [161,162]. In the systemic circulation, E2 via the HIF-1α–VEGF axis prevents/reduces endothelial dysfunction, vascular inflammation, and neointima formation [163,164]. Relevant to PAH, following endothelial damage, E2 plays a key role in promoting endothelial healing and angiogenesis [165].

### 7.1. Opposing Effects of E2 and 2ME on Angiogenesis (Key Role of HIF-1α)

Data coming from basic and clinical research of female cancers indicate that E2 and 2ME have opposite effects on angiogenesis (Figure 2A; previously reviewed in references [8]). The angiogenic properties of E2 are in striking contrast to the strong antiangiogenic effects of its major non-estrogenic metabolite 2ME. The other biological effects of 2ME relevant to PAH (Figure 2) have been reviewed previously [166].

The regulation of HIF1α activity is mechanistically linked to microtubules, and disruption of the microtubule cytoskeleton downregulates the HIF1-α pathway and tumor angiogenesis [167,168]. Similar to other tubulin disruptors, 2ME binds to (or near) the colchicine-binding site, disrupts the microtubule cytoskeleton, downregulates HIF1-α, and inhibits angiogenesis ([168]; Figure 2B). Notably, the most consistently reported effect of 2ME is its ability to inhibit HIF-1α. Indeed, this effect is so reliable that numerous recent studies have used 2ME as a pharmacological tool to inhibit HIF-α expression and signaling [167,168]. Furthermore, the use of 2ME has been proposed to combat HIF-signaling and related glycolytic shift in PAH [169]. Noteworthy, in vitro in swine granulosa cells, hypoxia stimulates 2ME production, which simultaneously inhibits hypoxia-driven angiogenesis [170], and in vivo, in a murine model of rheumatoid arthritis, 2ME reduces the expression of mRNA for the angiogenic cytokines and prevents neovascularization into the joint [171]. Thus, 2ME could be viewed as a local modulator of angiogenesis. In OVX CH-PH rats, 2ME inhibits oxidative stress-induced activation of the HIF-1α pathway [69,79], and similar effects are seen with E2. Whether this inhibitory effect of E2 on the HIF-1α pathway in CH-PH rats is mediated by E2′s downstream metabolite 2ME is presently unknown. Importantly, a recent study from the MacLean lab [76] confirmed the previously reported therapeutic effects of 2ME in hypoxic PH [77,78] suggested sex-dependent differences in HIF-1α signaling and supports the role of 2ME as an anti-angiogenic factor in PH [76]. In this study, basal HIF-1α protein expression was higher in female than male hPASMCs, and the antimitogenic effects of 2ME in hPASMCs were associated with reduced HIF-1α expression. Similarly, in vivo 2ME attenuated hypoxia-induced PH in male and female rats, while decreasing the protein expression of HIF-1α [76].

Presently, it is unclear whether the effects of E2 and 2ME on angiogenesis and vascular remodeling play a role in PAH. Whether, based on its angiogenic properties, E2 exhibits protective or injurious effects in the healthy or injured pulmonary vasculature is a subject of the ongoing debate regarding the estrogens paradox in angioproliferative PH. On this subject, the available data are limited to media remodeling and are contradictory. In the Su-Hx model, female sex is associated with reduced RV fibrosis and greater survival, but with greater media remodeling of the pulmonary arteries [49]. On the contrary, preventive E2 treatment in OVX Su + Hx rats attenuates media remodeling and prevents the development of PH [172]. Unfortunately, to date, no single study has analyzed the effects of E2 on occlusive and plexiform lesions in a Su-Hx model. 

### 7.2. 2ME, HIF1α, and Endothelial-to-Mesenchymal Transition in PAH

Hypoxia and HIF-1α upregulation drive endothelial-to-mesenchymal transition (EndoMT), i.e., ECs transition into a mesenchymal or myofibroblast phenotype. The activation of the HIF-1α pathway and subsequent EndoMT has been implicated in MCT- and CH-induced vascular remodeling and PH [173,174], as well as in the vascular pathology of pulmonary fibrosis and PAH in humans [175,176,177]. 2ME inhibits hypoxia- and irradiation-induced EndoMT and lung fibrosis via downregulation of HIF1α-dependent Smad signaling [178,179]. Whether 2ME reduces vascular remodeling via HIF-1α inhibition, not only in hypoxia-induced PH [76,77,78], but also in angioproliferative PH, needs to be determined.

### 7.3. 2ME, HIF-1α, and Metabolic Reprograming in PAH

A shift from oxidative phosphorylation to glycolysis (the Warburg effect) takes place in vasculature in PAH patients [168,180], and this shift has been linked to pathologic angiogenesis and pulmonary vascular remodeling. The Warburg effect has been investigated as a druggable target in highly proliferative ECs to reduce angiogenesis [181]. In this regard, the HIF-1α is a key regulator responsible for the glycolytic shift in vascular cells in both cancer and PAH patients [181,182]. HIF-1α-induced pyruvate dehydrogenase kinase (PDK) inhibits pyruvate dehydrogenase (PDH) and thereby increases glycolysis [180]. The PDK inhibitor dichloroacetate not only reverses the Warburg effect, but also has therapeutic effects on experimental PH [183,184,185]. The effects of estrogens on metabolic changes in pulmonary vasculature are unknown. Nonetheless, E2, via ERα-mediated upregulation of glycolytic enzymes, not only stimulates pathologic angiogenesis in breast cancer [186], but also boosts a mitogenic response in highly proliferative human endometrial cells and umbilical vein endothelial cells [187,188]. In contrast, because 2ME is a strong HIF-1α inhibitor, 2ME would be expected to inhibit metabolic reprograming in PAH. Although the effects of 2ME on metabolic reprograming in PAH are unknown, by inhibiting HIF-1α and PDK, 2ME attenuates glycolysis and inhibits proliferation of apoptosis resistant melanoma cells [189]. Whether, similar to cancer cells, 2ME inhibits metabolic reprograming in highly proliferative, apoptosis resistant endothelium in PAH warrants further investigation.

### 7.4. Future Directions and Clinical Implications

In contrast to solid experimental and clinical evidence about its beneficial effects on RV function, the role of E2 on angioproliferation (formation of occlusive and complex/plexiform lesions) in PAH remains largely unexplored. Future studies on the Su+Hx model should focus on head-to-head comparison of endogenous versus exogenous E2 and preventative versus rescue treatments with E2 and 2ME on metabolic reprograming and the development of occlusive and plexiform lesions in male and intact and OVX female animals.

## 8. Inflammation, Immunity, and Estradiol Metabolism in PAH

A growing body of evidence has implicated inflammation and altered immunity in the pathogenesis of PAH [190,191,192]. In PAH patients, the size of perivascular inflammatory and immune cell infiltrates correlates with intima + media remodeling and pulmonary artery pressure [193], and abnormally elevated levels of circulating proinflammatory cytokines are predictive of worse outcomes [194,195]. Notably, proinflammatory cytokines strongly induce aromatase and sulfatase activity [196,197] and peripheral E2 synthesis [198]. Because E2 upregulates CYP1B1 and sulfatase activity, in an inflammatory environment, via a feed-forward mechanism, E2 may increase the bioavailability of its metabolic precursor DHEA, which would augment its own production. In addition, upregulated CYP1B1 may increase the production of 4-HE and 16α-HE1, which are very estrogenic metabolites with significant angiogenic, proinflammatory, and mitogenic properties (Figure 3). As discussed above, increased E2 tissue levels may also augment the production of proinflammatory metabolites of arachidonic acid.

Women have stronger immune responses compared to men [199,200,201]. This may initially be physiologically beneficial, but with aging, a more aggressive immune response may become detrimental and may increase the risk of PAH. For example, ERα signaling promotes T cell activation and proliferation and contributes to T cell-mediated autoimmune inflammation [202]. Women more frequently develop various autoimmune diseases (including systemic sclerosis, systemic lupus erythematosus, mixed connective tissue disease, and rheumatoid arthritis) that are associated with increased risk of PAH [5]. The autoimmune inflammation may have significant effects on EMet and E2 levels. Both lung macrophages and immune cells express steroidogenic enzymes (sulfatase and aromatase), and in women with rheumatoid arthritis or systemic lupus, measures of autoimmune inflammation strongly correlate with E2 levels [203,204]. Furthermore, irrespective of sex, autoimmune inflammation augments the 16-hydroxylation pathway [205] and produces changes in sex steroids and their precursors and metabolites, similar to those reported in PAH [46,47,58].

### 8.1. Anti-Inflammatory and Immunomodulatory Effects of 2ME

In contrast to E2, non-estrogenic 2ME exhibits significant anti-inflammatory and immunomodulatory effects. The anti-inflammatory effects of 2ME are in part mediated by the suppression of macrophage activation [206,207], with the inhibition of macrophages influx/activation by 2ME reported in several models of cardiovascular and renal injury [208,209,210]. Furthermore, in an autoimmune model of rheumatoid arthritis, 2ME inhibits the expression of mRNA for inflammatory cytokines (IL-1β, TNF-α, IL-6, and IL-17), reduces local inflammation and prevents neovascularization and disease progression [171]. Furthermore, in several models of autoimmune inflammatory disease, 2ME attenuates the progression of disease by inhibiting T cell activation, proliferation, and cytokine release [211,212,213]. Of significance to PH, 2ME, its metabolic precursor 2HE, its “inactive” metabolite 2ME1, and/or its synthetic analog 2-ethoxyestradiol reduce the influx/activation of macrophages (ED1+ cells) in MCT- and bleomycin-induced PH, which correlates with reduced right ventricular peak systolic pressure and vascular remodeling and fibrosis [12,20,21,22,79,113,114,115,116,117,118]. 

### 8.2. Future Directions and Clinical Implications

An inflammatory environment and altered immunity may have significant effects on E2 metabolism. Through multiple mutually non-exclusive mechanisms, autoinflammation may increase the bioavailability of E2, other proinflammatory “bad estrogens”, and proinflammatory AA metabolites. As discussed above, the reported hormonal changes in men and postmenopausal women with PAH are similar to changes seen in autoimmune inflammatory diseases (systemic sclerosis and lupus). This suggests that, irrespective of sex and type of immune disease (Th1 versus Th2), the autoinflammation is associated with increased activity of sulfatase, aromatase, and 17β–HSD. Future translational and clinical studies should assess the impact that inflammation and altered immunity have on key metabolizing enzymes and E2 and AA metabolism in PAH.

## 9. Concluding Remarks and Future Directions

**Figure 5:**
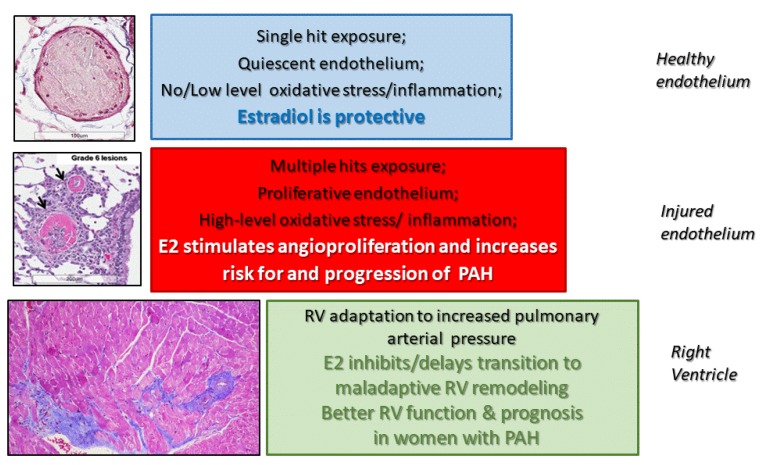
Three-tier effects of estradiol concept in PAH. The effects on vascular pathobiology, progression, and prognosis of PAH are shown. Estradiol could be viewed as: (1) the protector of heathy pulmonary vasculature; (2) the instigator and perpetuator of disease in injured pulmonary vascular; and (3) the protector of the overloaded right ventricle.

The proposed three-tier concept of estrogen action in PAH (Figure 5) offers an explanation for the contradictory effects of estrogens reported in different models of PH and in men and women with PAH.

Estrogens could be viewed as protectors of RV function in PAH, yet they can be considered instigators and perpetuators of vascular injury in the pulmonary circulation, which lead to the development of PAH. Based on the reported cellular effects and limited animal data, we view 2ME as a biological antagonist of E2 at the level of pulmonary ECs and AA metabolism. 2ME, via a multitude of actions (Figure 2 and Figure 3), can not only provide pulmonary vascular and RV protection, but also oppose the potential detrimental effects of E2 due to redirection of its own metabolism and metabolism of AA in PAH. Thereby, 2ME could be considered as both mediator and corrector of E2′s action and a new disease modifier in PAH [166].

Recently applied combination therapies, attacking different pathogenic pathways in PAH, have brought additional benefits to PAH patients over mono therapies [214,215]. Currently, three ongoing clinical trials are assessing the therapeutic benefits of inhibition of E2 production (anastrazole) or E2 signaling (tamoxifen and fulvestrant) in PAH patients. Testing combinations of modulators of E2 metabolism in experimental PH may give additional insight into the role of E2 metabolism in PAH and open the venue for clinical testing of E2 metabolites and metabolism modulators in both women and men with PAH. Combination therapy of E2 metabolism modulators/metabolites may bring more subtle and targeted correction of E2 metabolism derangements in PAH and offer protective and therapeutic effects in both women and men with PAH.

A multitude of factors and conditions can affect E2 metabolism (inflammation, hypoxia/oxidative stress, obesity/insulin resistance/leptin, diet, drugs, and genetic polymorphisms of metabolizing enzymes), suggesting that PAH heterogeneity may be due, at least in part, to dysregulated E2 metabolism. Increased E2 and decreased 2ME production may adversely affect pulmonary vascular remodeling and the progression of disease. Therefore, in-depth investigation of the vascular and RV effects of more than a dozen biologically active metabolites in severe PAH is warranted. Future prospective, randomized clinical trials (using highly selective and sensitive mass spectrometry-based methods) should not only quantify multiple estrogens, their metabolites, and metabolic precursors, but also markers of inflammation and angiogenesis. The use of high-throughput molecular data and contemporary computational techniques focused on the E2 metabolome may identify different PAH phenotypes and foster the precision medicine approach in PAH. In PAH patients, an accurate assessment of the activity of the numerous enzymes involved in estrogen metabolism may be very difficult. Animal and cell culture studies focused on sex hormone intracrinology (i.e., intracellular disposition and intracellular/extracellular equilibrium) in pulmonary vascular and RV cells would augment our understanding of the role of E2 metabolism in the inflammation, angioproliferation, and RV dysfunction of PAH.

## Figures and Tables

**Table 1:** Effects of sex hormones, their precursors, and metabolism inhibitors experimental PH and human PAH.

Animal Model PAH Patients	Hormone, Sex, Enzyme, Treatment	Effects/Comments	References
CH- and MCT-PH in rats	Estradiol (E2)	Female sex protective; E2 attenuate PH and RV and vascular remodeling; OVX exacerbates PH	Reviewed previously in Tofovic SP, 2010 [8]
MCT female and OVX rats	Estradiol	E2 attenuates whereas OVX exacerbates PH; ↓ E2 levels and aromatase, ↑ Cyp 1A1 and Cyp1B1	Yuan P. et al., 2013 [48]
Su + Hx male and female rats	Female versus Male	Female: greater pulmonary vascular remodeling; Male: more severe RV failure, lower survival rate	Rafikova et al., 2015 [49]
Chronic hypoxia (CH)- induced PH in rats	DHEA	Prevents or reverses PH; Inhibits PH and augments vasodilator responsiveness to NO	Bonnet S. et al., 2003; Oka M. et al., 2007 [37,39]
MCT + pneumonectomy rat	DHEA	Rescue treatment attenuates PH and vascular remodeling and eliminates late mortality	Homma N. et al., 2008 [40]
MCT rat	DHEA	Reduces PH and RV hypertrophy	Paulin R. et al., 2011 [41]
Su + Hx rat	DHEA	Attenuates PH; restores RV structure/function	Alzoubi A. et al., 2012 [44]
CH-PH rats Rat-pup model of CH-PH	DHEA	Reverses CH/reoxygenation-induced RV dysfunction; Attenuates PH, RV, and vascular remodeling in infant rats	Dumas de La Roque E. et al., 2012; 2013 [43,50]
Pregnant Sheep	DHEA	Vasodilator effects on fetal pulmonary circulation	Sharma D. et al., 2018 [42]
Rat model of left heart failure (LHF)-induced PH	DHEA	Attenuates LHF-induced PH and RV and vascular remodeling	Zhang YT. et al., 2019 [51]
PM women, idiopathic, CTD- or CHD-PAH	Low DHEA-S High E2	Increased risk and severity of PAH in postmenopausal women	Baird GL et al., 2018 [47]
Men with idiopathic, heritable, or CTD-PAH	Low DHEA-S High E2	Associated with risk of PAH; ↑ E2, shorter 6MWD; ↑ DHEA-S, lower right atrial pressure	Ventetuolo CE et al., 2016 [46]
Patients with COPD	DHEA	Improves PH in COPD patients	D. La Roque E. et al., 2012 [45]
Men with idiopathic PAH	Estradiol Testosterone (T) Progesterone (P)	↑ E2 and E2/T ratio and ↓ T and P associated with ↑ risk of PAH; high E2 independently associated with higher mortality	Wu W-H. et al., 2018 [52]
Premenopausal women with idiopathic PAH	FSH Progesterone	↑ FSH and ↓ P tended to be associated with high risk of IPAH and mortality among patients	Zhang Y-X et al., 2019 [53]
Su+Hx female rats	Anastrozole	Reduces PH and number of occlusive and PLX lesions, but has no effect on RV remodeling.	Tofovic SP. et al., 2013 [54] *
CH mice and Su+Hx rats	Anastrozole	Attenuates PH only in female animals	Mair KM. et al., 2014 [55]
Su+Hx rats	Metformin	↓ circulating estrogens and aromatase levels/activity, and attenuates PH and vascular and RV remodeling	Dean A. et al., 2016 [56]
BMPR2 mutant mice	Anastrozole + Fulvestrant	Prevents and Reverses PH and reduces BMPR2 mutation associated with metabolic defects	Chen X. et al., 2017 [57]
Portopulmonary hypertension	Estradiol Aromatase	Irrespective of gender, in patients with liver disease, ↑ aromatase and ↑ E2 levels increase risk of portopulmonary PH	Roberts KE. et al., 2009 [58]
PAH patients	Anastrozole	Reduces serum E2 and E1 levels, significantly increases 6MWD, but has no effect on RV function	Kawut SM et al., 2017 [59]
Su + Hx OVX female rats	Estradiol	Protects RV function and restores RV ventricular–vascular coupling efficiency	Liu A. et al., 2017 [60]
Su + Hx male, female and OVX rats	Estradiol	Endogenous and exogenous E2 exerts protective effects on baseline RV function and after an acute exercise challenge	Frump, AL. et al., 2015 [15] Lahm T. et al., 2016 [13]
Su + Hx female and OVX mice	Estradiol	E2 has no effect on PH, yet reduces proximal conduit arteries stiffness and improves ventricular–vascular coupling; no data on RV remodeling available	Liu A. et al., 2015 [61]
Su + Hx female and OVX mice	Estradiol	In absence of RV remodeling in diseased animals, improves RV function (enhances RV contractility in response to PH and preserves cardiac reserve)	Liu A. et al., 2014 [62]
MCT in Apo E deficient female mice	Estradiol	Rescue treatment reduces PH and RV hypertrophy	Umar S. et al., 2017 [63]
Female and OVX mice overexpressing SERT	Estradiol	Only female, SERT+ mice develop PAH, OVX abolishes and E2 treatment of OVX + SERT mice reestablishes PH.	White K. et al., 2011 [64]
MCT OVX rats	Progesterone	Attenuates PH, and RV and vascular remodeling	Tofovic SP. et al., 2009 [65]

↑—increased; ↓—reduced; RV—right ventricle; PM—postmenopausal; OVX—ovariectomy; CTD—connective tissue disease; CHD—congenital heart disease; SERT—serotonin transporter; *—published in form of abstract.

**Table 2:** Effects of estradiol metabolites in PH.

Animal Model	Estradiol Metabolite	Effects Comments	Reference
MCT male rats	2-methoxyestradiol 2-hydroxyestardiol	Attenuate development or progression of PH	Tofovic SP et al., 2005 [12]
MCT OVX and female rats	2-methoxyestradiol	Ovariectomy exacerbates PH, whereas 2ME attenuates PH in OVX rats; no estrogenic effects	Tofovic SP et al., 2006 [26]
MCT male rats	2-ethoxyestradiol	Synthetic metabolite; attenuates PH, lung inflammation, and RV and vascular modeling, and eliminates late mortality	Tofovic SP et al., 2008 [22]
Bleomycin-induced PH and lung fibrosis, OVX female rats	2-methoxyestradiol	Ovariectomy exacerbates whereas 2ME attenuates PH, inflammation, fibrosis and vascular remodeling	Tofovic SP et al., 2009 [21]
MCT male rats	2-methoxyestradiol	Dose-dependent therapeutic effects on PH, lung inflammation and RV and vascular remodeling; no estrogenic effects	Tofovic SP et al., 2010 [113]
MCT male rats	2-methoxyestradiol	Synergistic effects with bosentan or sildenafil on amelioration of PH, lung inflammation, and vascular remodeling	Tofovic SP et al., 2010 [12]
CH male and female rats	2-methoxyestradiol	Attenuates development and retards the progression of PH; decreases HIF-1α expression and reduces elevated hematocrit	Wang L. et al., 2017; Hao S. et al., 2019 Docherty CK. et al., 2019, Tofovic SP. et al., 2005 * [77,78,79,114]
MCT male rats	2-methoxyestradiol +/− retinoic acid	Synergistic effects of 2ME with retinoic acid on amelioration of PH	Tofovic SP et al., 2008 [115] *
MCT OVX rats; Female and OVX Su+Hx rats	2-methoxyestrone	Attenuates MCT-induced PH, inflammation and RV and vascular remodeling; attenuates Su+Hx-induced PH, RV dysfunction, and remodeling and number of occlusive lesions	Tofovic SP. et al., 2008, Hu J. et al., 2016 [116,117] *
Su+Hx OVX rats	4-hydroxyestradiol	Has no effect on PH, but attenuates RV hypertrophy	Tofovic SP. et al., 2013 [54] *
Obese ZDSD rats	2-hydroxyestradiol	Reduces HbA1c and attenuates metabolic syndrome-induced PH in male rats	Tofovic SP. et al., 2014 [118] *
Female mice	16α - hydroxyestrone	Induces mild PH and RV and vascular remodeling	White K. et al., 2012 [119]
BMPR2 mutant mice	16α -hydroxyestrone	Exacerbates BMPR2-associated PH	Chen X. et al., 2016 [120]

*—Published in form of abstract; MCT—monocrotaline; CH—chronic hypoxia. HbA1c-Glycosilated hemoglobin; BMPR2-bone morphogenetic protein receptor type 2.
